# Supplementary material for: A Likelihood Approach for Real-Time Calibration of Stochastic Compartmental Epidemic Models
Source: PLoS Comput Biol. 2017 Jan 17;13(1):e1005257. doi: 10.1371/journal.pcbi.1005257 (PMC5240920; doi:10.1371/journal.pcbi.1005257)
Supplement: S1 File — (TAR.GZ) [file pcbi.1005257.s014.tar.gz › HSPH_Online-SI-Revision/IPoi09/n10-severe/n10-severe_BENCH_summary.pdf]

peak -8	R0	$1/\theta_2$	inf	inflog	newly	newlylog	Reff	AttackRate
mean	29.3	71.4	233.3	0.5	265.	0.6	32.9	37.7
median	26.8	63.1	132.5	0.4	160.4	0.5	30.9	36.8
Quantile .1	8.2	28.	73.3	0.3	75.1	0.4	13.8	16.9
Quantile .9	53.1	119.1	279.4	0.8	358.1	1.	55.8	61.5
26	19	27						
length	50							

peak -4	R0	$1/\theta_2$	inf	inflog	newly	newlylog	Reff	AttackRate
mean	28.4	65.6	144.3	0.4	91.2	0.3	28.7	26.4
median	21.6	42.8	137.	0.4	86.	0.3	24.7	24.8
Quantile .1	3.9	14.5	58.8	0.2	43.8	0.2	15.	7.2
Quantile .9	60.	152.2	247.8	0.5	153.1	0.4	48.2	50.2
3	8	7						
length	50							

peak	R0	$1/\theta_2$	inf	inflog	newly	newlylog	Reff	AttackRate
mean	67.9	128.1	198.7	0.5	44.9	0.2	40.1	47.3
median	64.5	104.3	177.5	0.4	45.3	0.2	39.9	45.3
Quantile .1	49.	60.8	116.	0.3	20.8	0.1	33.3	34.6
Quantile .9	93.4	211.1	316.	0.6	71.4	0.2	47.4	62.7
0	0	0						
length	50							

peak +4	R0	$1/\theta_2$	inf	inflog	newly	newlylog	Reff	AttackRate
mean	72.9	139.3	394.1	0.6	109.5	0.3	47.9	49.3
median	73.6	123.3	286.2	0.5	89.1	0.2	48.6	47.2
Quantile .1	51.3	80.5	165.8	0.4	51.2	0.2	39.1	34.8
Quantile .9	93.	212.8	628.5	0.8	161.7	0.4	55.5	67.5
0	0	0						
length	50							

peak +8	R0	$1/\theta_2$	inf	inflog	newly	newlylog	Reff	AttackRate
mean	69.9	129.5	960.2	0.7	300.7	0.4	54.1	48.6
median	71.9	119.9	398.5	0.6	154.7	0.3	54.5	47.2
Quantile .1	50.3	80.5	214.9	0.4	72.6	0.2	44.	34.5
Quantile .9	84.7	190.2	1239.8	1.	449.	0.7	64.6	64.6
0	0	0						
length	50							
